# Carbon monoxide targeting mitochondria in astrocytes: modulation of cell metabolism, redox response and cell death

**DOI:** 10.1186/2193-1801-4-S1-L42

**Published:** 2015-06-12

**Authors:** Helena Vieira, Ana Almeida, Cláudia Figueiredo-Pereira, Cláudia Queiroga

**Affiliations:** Chronic Diseases Research Center, NOVA Medical School, Universidade Nova de Lisboa, Portugal

**Keywords:** carbon monoxide, astrocytes, mitochondria

The endogenously produced gasotransmitter carbon monoxide (CO) has been studied as a factor involved in cytoprotection, homeostasis and anti-inflammation. Small amounts of reactive oxygen species (ROS) are described as signaling factors in CO’s biological mode of action. Mitochondria are the main source of ROS and are also key organelles in orchestrating cell function: metabolism, cell death control and redox signaling. Astrocytes are most abundant glial cells and essential for neuronal function, namely metabolic and physical support, expression of neurotransmitters and promotion of neuroprotection. In this work it is shown that CO prevents astrocytic cell death and improves cell metabolism by targeting mitochondria, and some of the underlying molecular mechanism are disclosed. CO directly targets non-synaptic mitochondria and inhibits their mitochondrial membrane permeabilization, by preventing mitochondrial swelling, depolarization and inner membrane permeabilization. Thus, CO limits the release of cytochrome c into the cytosol and the activation of apoptotic cascade in astrocytes. All these events are ROS-dependent and involve glutathionylation of adenine nucleotide translocator (ANT), whose activity is ATP/ADP transport through mitochondrial inner membrane. In addition, low amounts of exogenous CO increase ATP production by improving oxidative metabolism. Mitochondrial population and specific cytochrome c oxidase activity are higher upon CO treatment. The CO-induced metabolic improvement is dependent on Bcl-2 expression. Dysfunctional mitochondrial can be eliminated by mitophagy, which is a crucial process for maintaining their function and quality control. In astrocytes, CO promotes mitophagy at 1h of treatment, while following 24h mitochondrial population is back to basal levels, indicating that CO contributes to mitochondrial turnover. Furthermore, CO limits astrocytic cell death in an autophagic dependent manner.

